# Supplementary figures and images for: Positive allosteric modulation of emodepside sensitive Brugia malayi SLO-1F and Onchocerca volvulus SLO-1A potassium channels by GoSlo-SR-5-69
Source: PLoS Pathog. 2025 Sep 11;21(9):e1012946. doi: 10.1371/journal.ppat.1012946 (PMC12440207; doi:10.1371/journal.ppat.1012946)

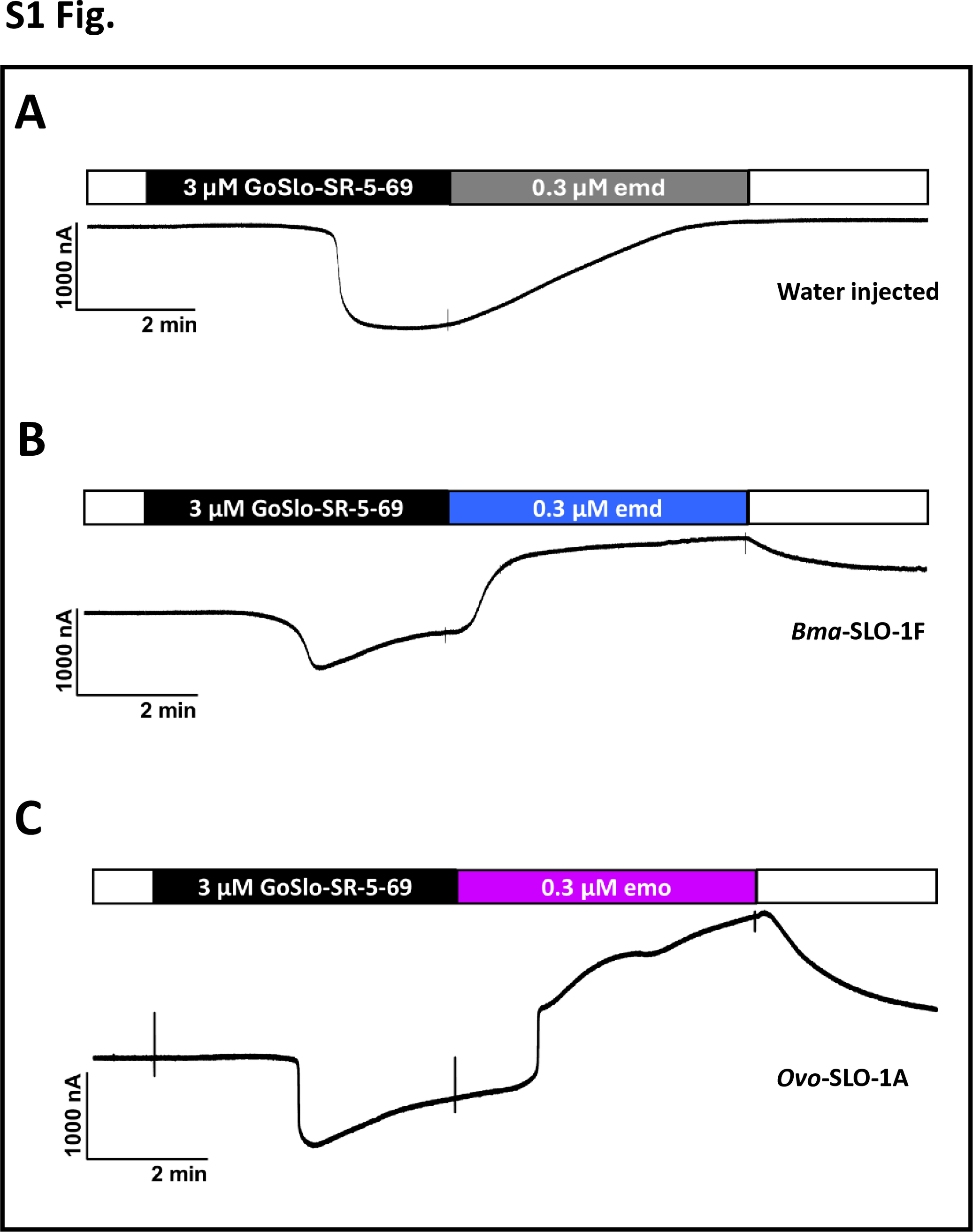

Supplement: S1 Fig — A. Representative trace of water injected oocytes. B. Representative trace of oocytes expressing the Bma-SLO-1F channel. C. Representative trace of Ovo-SLO-1A expressing channel. Oocytes were recorded at a steady-state potential of +20 mV. (TIF) [file ppat.1012946.s001.tif]

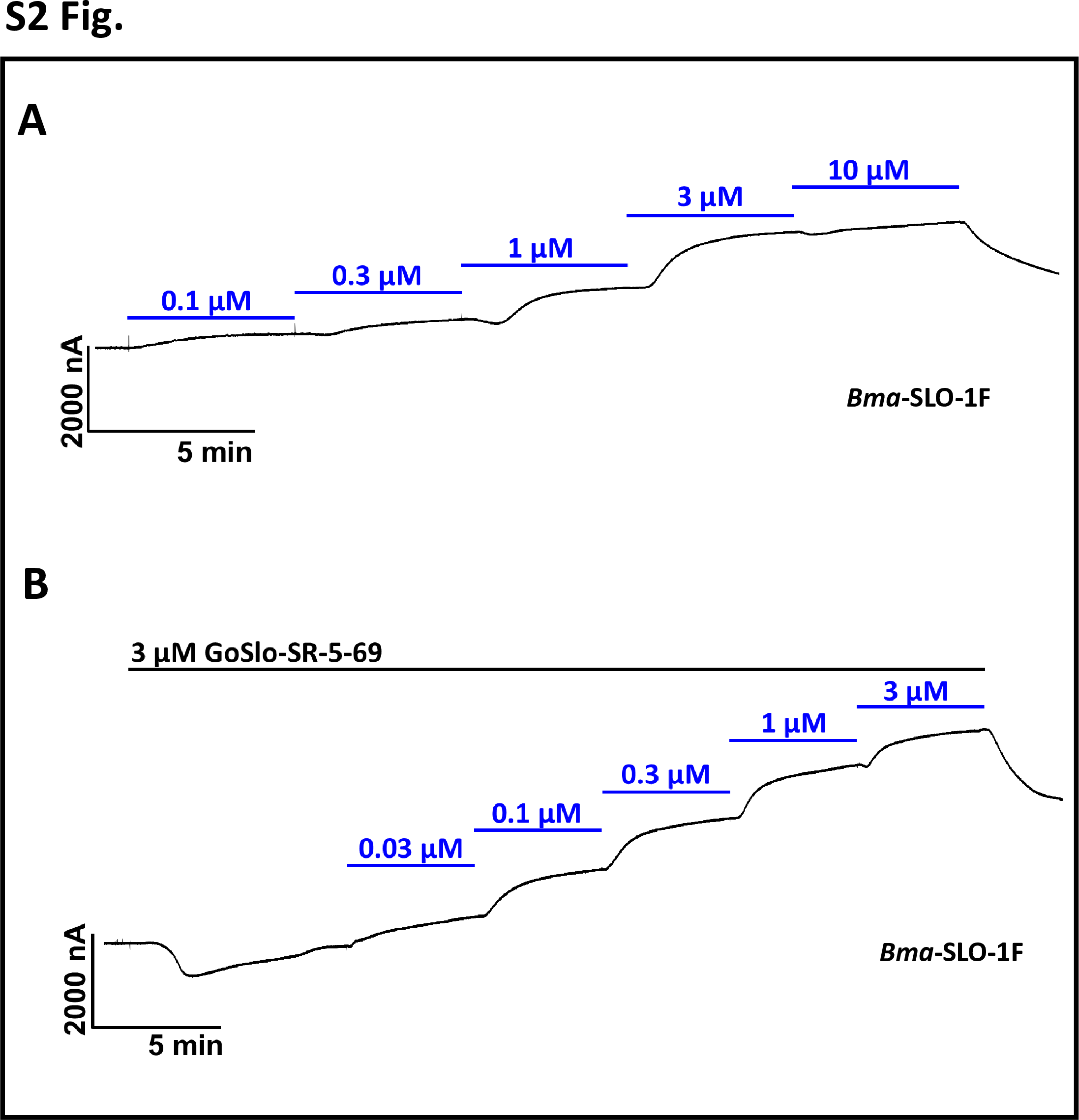

Supplement: S2 Fig — A. Representative current traces for two-electrode voltage-clamp recording showing outward currents for Bma-SLO-1F in response to increasing concentrations of emodepside (0.1 to 10 µM) at a steady-state holding potential of +20 mV. B. Representative current traces for two-electrode voltage-clamp recording showing outward currents for Bma-SLO-1F in response to increasing concentrations of emodepside (0.1 to 10 µM) in the presence of 3 µM GoSlo-SR-5–69 at a holding potential of +20 mV. (TIF) [file ppat.1012946.s002.tif]

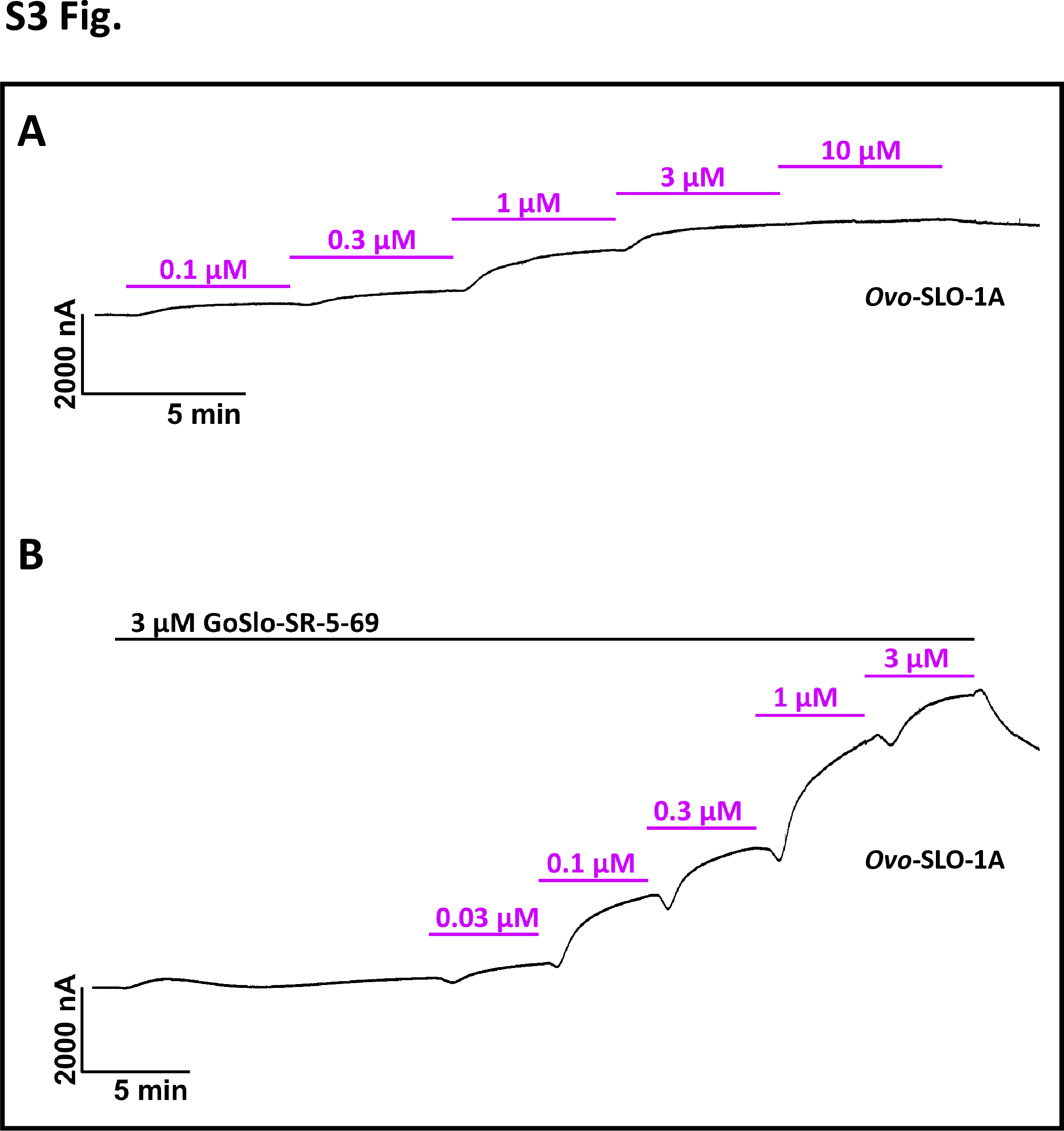

Supplement: S3 Fig — A. Representative current traces for two-electrode voltage-clamp recording showing outward currents for Ovo-SLO-1A in response to increasing concentrations of emodepside (0.1 to 10 µM) at a steady-state holding potential of +20 mV. B. Representative current traces for two-electrode voltage-clamp recording showing outward currents for Ovo-SLO-1A in response to increasing concentrations of emodepside (0.1 to 10 µM) in the presence of 3 µM GoSlo-SR-5–69 at a holding potential of +20 mV. (TIF) [file ppat.1012946.s003.tif]

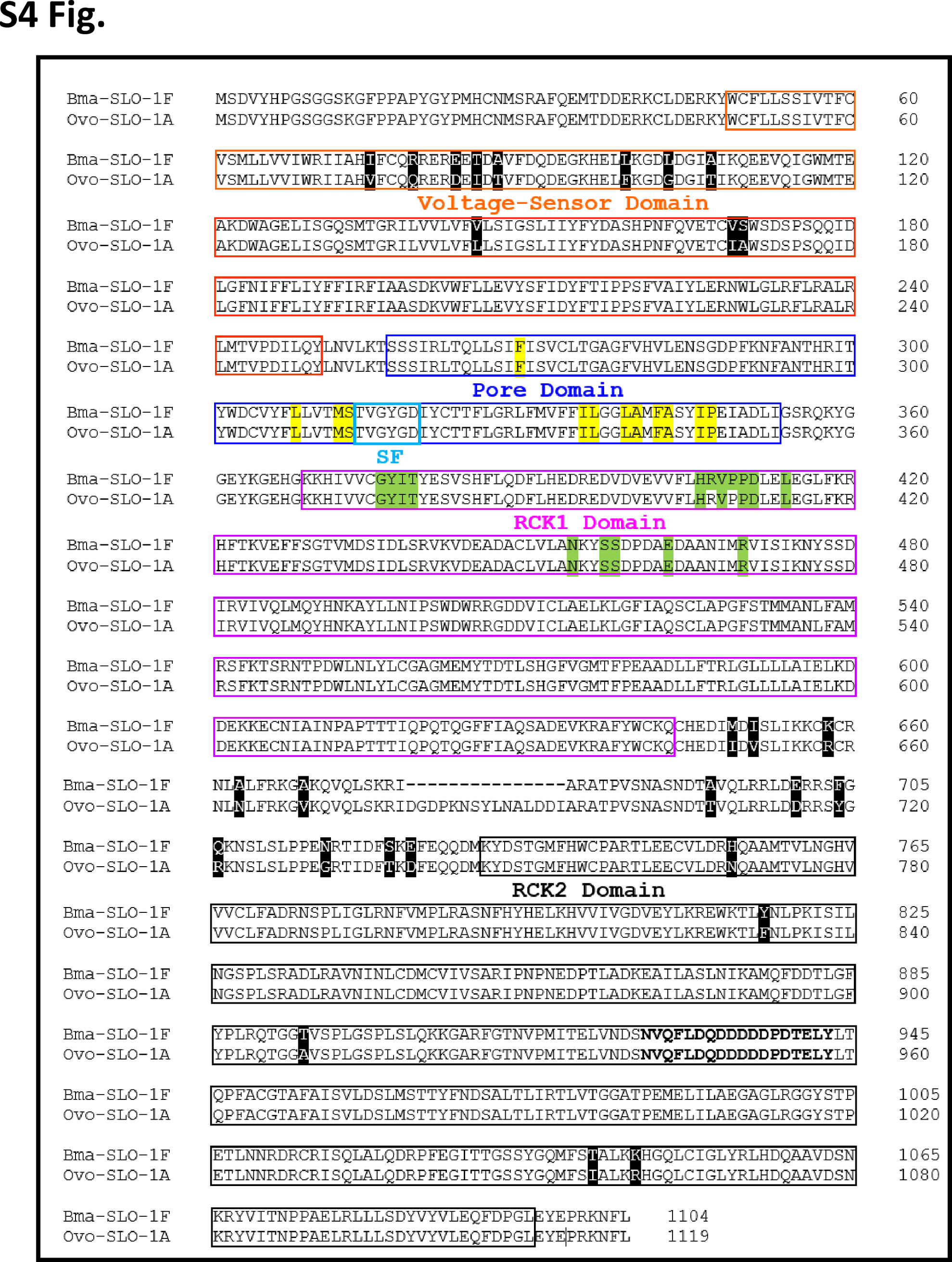

Supplement: S4 Fig — The voltage sensor domain (VSD; orange boxes), pore domain (PD; blue boxes) comprising the selectivity filter (light blue box), and two C-terminal domains for regulator of K+ conductance (RCK1; pink boxes and RCK2; black boxes) are indicated. Amino acids which are not identical between filarial species are highlighted by a black background. Gaps are indicated by “_” symbols for amino acid residues that are missing. Residues that are predicted to be involved in emodepside binding are highlighted by a yellow background in the PD. Putative amino acid residues involved in GoSlo-SR-5–69 binding are highlighted by a light green background in the RCK1 domain. Note that both Bma-SLO-1F and Ovo-SLO-1A have conserved amino acids interacting with GoSlo-SR-5–69 except for R407 and P409 that are not involved in binding for Ovo-SLO-1A. (TIF) [file ppat.1012946.s004.tif]
